# Sub-nanosecond tryptophan radical deprotonation mediated by a protein-bound water cluster in class II DNA photolyases†
†Electronic supplementary information (ESI) available: For Fig. S1–S7 and Scheme S1. See DOI: 10.1039/c7sc03969g


**DOI:** 10.1039/c7sc03969g

**Published:** 2017-12-11

**Authors:** Pavel Müller, Elisabeth Ignatz, Stephan Kiontke, Klaus Brettel, Lars-Oliver Essen

**Affiliations:** a Institute for Integrative Biology of the Cell (I2BC) , CEA , CNRS , Univ. Paris-Sud , Université Paris-Saclay , 91198 , Gif-sur-Yvette cedex , France . Email: pavel.muller@i2bc.paris-saclay.fr; b Department of Chemistry , LOEWE Center for Synthetic Microbiology , Philipps University , 35032 Marburg , Germany . Email: essen@chemie.uni-marburg.de

## Abstract

Class II DNA photolyases are flavoenzymes occurring in both prokaryotes and eukaryotes including higher plants and animals. Despite considerable structural deviations from the well-studied class I DNA photolyases, they share the main biological function, namely light-driven repair of the most common UV-induced lesions in DNA, the cyclobutane pyrimidine dimers (CPDs). For DNA repair activity, photolyases require the fully reduced flavin adenine dinucleotide cofactor, FADH^–^, which can be obtained from oxidized or semi-reduced FAD by a process called photoactivation. Using transient absorption spectroscopy, we have examined the initial electron and proton transfer reactions leading to photoactivation of the class II DNA photolyase from *Methanosarcina mazei*. Upon photoexcitation, FAD is reduced *via* a distinct (class II-specific) chain of three tryptophans, giving rise to an FAD˙^–^ TrpH˙^+^ radical pair. The distal Trp_388_H˙^+^ deprotonates to Trp_388_˙ in 350 ps, *i.e.*, by three orders of magnitude faster than TrpH˙^+^ in aqueous solution or in any previously studied photolyase. We identified a class II-specific cluster of protein-bound water molecules ideally positioned to serve as the primary proton acceptor. The high rate of Trp_388_H˙^+^ deprotonation counters futile radical pair recombination and ensures efficient photoactivation.

## Abstract 2

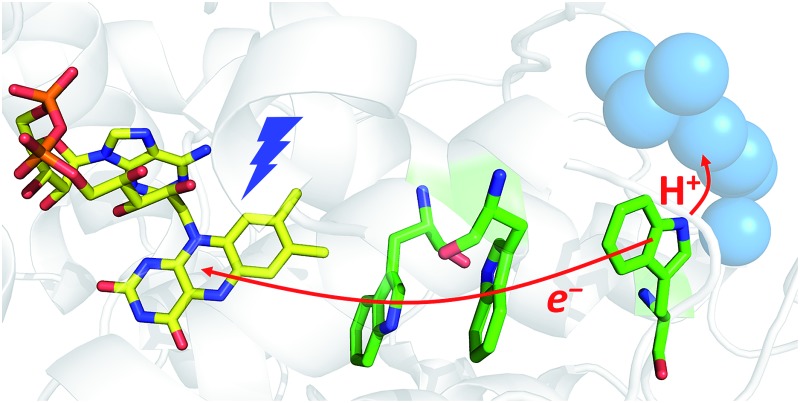
Light activation of class II DNA photolyases is enhanced by a unique cluster of protein-bound water molecules.

## Introduction

The exposure of DNA to UV light causes serious damage of the genetic code and eventually results in fatal mutations. The most prominent forms of UV-induced lesions are cyclobutane pyrimidine dimers (CPDs) and the pyrimidine(6-4)pyrimidone photoproducts ((6-4)PPs). Several mechanisms evolved to restore the integrity of DNA, like nucleotide excision repair, base excision repair and photorepair.^1^,^2^ The latter is catalysed by photolyases, a class of flavoenzymes, which belong to the photolyase/cryptochrome-superfamily (PCSf).^3^ Photolyases are substrate-specific and they are divided into (6-4) and CPD photolyases, which are further subdivided into classes I–III. Compared to other members of the PCSf, the class II CPD photolyases, which occur in plants, animals and many microbial organisms, are highly divergent in terms of their sequences, especially in functionally important parts of the catalytic C-terminal domain.^4^–^6^ Cryptochrome (Cry) blue light receptors of plants and animals evolved from class I CPD and (6-4) photolyases, respectively, but they have mostly lost the ability to repair DNA. They are involved in multiple light-regulation processes^3^ and possibly also in the light-dependent ‘magnetic compass’ of migrating birds and certain other animals.^7^,^8^

For DNA repair, all photolyases depend on a fully reduced FAD cofactor, FADH^–^. Upon photoexcitation, FADH^–^ transfers an electron to the damaged DNA and thereby catalyzes the repair of the lesion. Isolated photolyases often contain a semi-reduced (FADH˙) or even a fully oxidized flavin (FAD_ox_).^4^,^9^–^11^

Photocatalytically active FADH^–^ is generated from oxidized flavin species by a second light-induced reaction, usually referred to as photoactivation. In the case of initially oxidized FAD_ox_, the FAD˙^–^ resulting from the primary photoinduced electron transfer (ET) becomes protonated (on a timescale of a few hundred milliseconds to a few seconds) to form FADH˙.^12^,^13^ Further reduction of FADH˙ to FADH^–^ can be achieved by absorption of another photon, inducing transfer of a second electron to the flavin cofactor.

The mechanism of photoactivation has been studied in detail *in vitro* for a class I CPD photolyase (from *E. coli*) and for a (6-4) photolyase (from *X. laevis*).^12^–^18^ Upon photoexcitation, FAD_ox_ or FADH˙ abstract an electron from the first member of a chain of three^14^ (or four^12^) tryptophan residues to form a primary FAD˙^–^ TrpH˙^+^ radical pair (in 0.5 to 0.8 ps)^17^,^18^ or an FADH^–^ TrpH˙^+^ pair (in ∼30 ps),^14^ respectively. The electron hole then migrates along the Trp chain from the flavin-nearest Trp towards the most distant one, stabilizing the pair thermodynamically and by its spatial separation within less than 100 picoseconds.^16^–^18^ Further stabilization of the pair is achieved by deprotonation of the exposed terminal TrpH˙^+^ cation radical by the solvent, which typically occurs within a few hundreds of nanoseconds.^12^,^14^,^19^ Finally, the resulting Trp˙ radical is scavenged by an extrinsic reducing agent. Similar light-induced electron and proton transfer reactions were observed in Crys.^20^

**Fig. 1:**
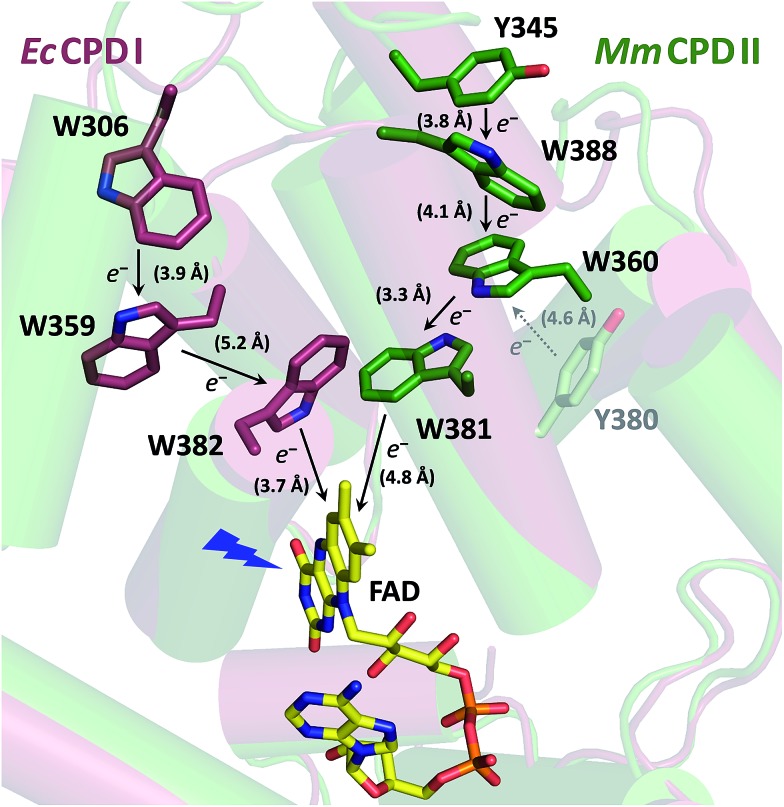
Superposition of the crystal structures from *Ec*CPDI (PDB entry ; 1DNP) and *Mm*CPDII (PDB entry ; 2XRZ) illustrating the different localization of the tryptophan cascades for electron transfer to FAD. The numbers in brackets indicate the shortest edge-to-edge distances in angstroms.

Despite the low sequence identity with other photolyases and cryptochromes (<16%),^21^ class II CPD photolyases such as the one studied here – *Mm*CPDII from the archaeon *Methanosarcina mazei* – share the same overall structural fold, with an N-terminal domain comprising a Rossman-like fold and an α-helical C-terminus which harbors the catalytically active FAD cofactor.^4^ Interestingly, class II CPD photolyases differ from other branches of the PCSf by the localization of the tryptophan cascade and by the presence of auxiliary tyrosine residues (Fig. 1). A previous mutational study of this ET pathway revealed that the Trp triad can also be functional as a dyad including only the first two FAD-proximal tryptophans.^4^

In this study, we investigated the photoreduction of FAD_ox_ to FADH˙ *via* FAD˙^–^ in *Mm*CPDII photolyase using transient absorption spectroscopy. We identified a cluster of water molecules involved in an unprecedentedly fast deprotonation of the distal tryptophan, Trp_388_. Additionally, we demonstrated that a conserved tyrosine, Tyr_345_, also participates in electron transfer to photoexcited FAD. This work represents the first endeavor to thoroughly characterize the initial photoactivation step in a class II CPD photolyase.

## Experimental

### Multiple sequence alignment-based analysis of class II CPD photolyases

451 non-redundant class II sequences with a pairwise sequence identity of less than 90% were extracted from a previous sequence-similarity network analysis of the photolyase-cryptochrome superfamily (combined PFAM protein families PF00875 and PF04244)^22^ using the Cytoscape suite.^23^ The multiple sequence alignment was done using Clustal omega.^24^ WebLogo^25^ was used for the visualization of the degree of position-specific conservation.

### Protein preparation

#### Cloning of *Mm*CPDII mutants

The generation of the E387Q mutant was based on pET-28a-*Mm*CPDII and done according to the phusion protocol (NEB) using the 5′-phosphorylated primers listed below. The preparation of the W388F and Y345F mutants was described earlier.^4^

E387Q primer

Sense: 5′-Pho-CAG TGG AGC GAA TCT CCC GAA AAA-3′

Reverse: 5′-Pho-CAG AAT TTT TTT TGC CCA GTA CAT GCG-3′

#### Overexpression and purification of *Mm*CPDII and mutants

Overexpression of *Mm*CPDII and mutants was done as described previously using *E. coli* BL21-Gold(DE3) cells (Stratagene).^4^ The cultivation was done in terrific broth medium for 24 hours at 25 °C (20 °C for W388F). The proteins were purified using a NiNTA column (MACHEREY-NAGEL) with 50 mM NaH_2_PO_4_, 300 mM NaCl, pH 8.0 and SEC column with Superdex 200 material (GE-Healthcare) with 10 mM Tris–HCl of pH 8.0 and 100 mM NaCl.

### Experimental conditions

Unless otherwise stated, the solutions of wild-type (WT) and all mutant *Mm*CPDII photolyases studied herein contained 10 mM Tris–HCl buffer at pH 8.0 (measured at room temperature), 100 mM NaCl and 10% (v/v) glycerol. For the experiment in D_2_O, 10 mM Tris–HCl buffer (at pH 8.0) with 100 mM NaCl was lyophilised to dry powder and the sublimated H_2_O was replaced by D_2_O, adding up to the original volume of the H_2_O buffer; note that both H_2_O and D_2_O samples in this experiment (Fig. 5) were hence glycerol-free. In the experiments where cysteine was added as an external reducing agent, cysteine was first dissolved in a more concentrated Tris–Cl buffer with NaCl. After titration by NaOH back to pH 8.0 and the addition of glycerol and water to the desired final volume, a stock of 500 mM cysteine solution was finally obtained in the standard 10 mM Tris–Cl buffer with 100 mM NaCl and 10% (v/v) glycerol. The addition of cysteine to the photolyase sample has hence diluted the protein but the concentrations of other components (buffer, salt and glycerol) and the pH were kept constant. Cysteine was chosen rather than the more commonly used reductant dithiothreitol because the latter was shown to be less efficient in a similar system and to cause protein precipitation at high concentrations.^26^

All samples were air-saturated and kept at 7 °C during the measurements or on ice in between. Before each experiment, they were rid of free FAD and other low-molecular-weight impurities by filtration over size-exclusion columns (Micro Bio-Spin, Bio-Gel P-6). The UV/vis spectrum was checked before and after each measurement to ensure that the sample was in a good shape, *i.e.*, not aggregated and FAD was not released, but protein-bound and fully oxidized (>95% FAD_ox_). UV/vis spectra were recorded on a Uvikon XS spectrometer (Secomam).

### Transient absorption spectroscopy

Transient absorption kinetics were measured on three different setups described in detail in ref. 12, 13, 19 and 27.

In experiments on ps/ns timescales, the photolyase samples were excited at 355 nm by a Nd:YAG laser (Continuum Leopard SS-10, pulse duration of 100 ps, repetition rate 1 or 2 Hz, and an energy in the order of 5 mJ per cm^2^).

In all other experiments, the samples were excited at 470 nm by laser flashes of 5 ns duration and an energy ≤10 mJ cm^–2^, delivered by a Nd:YAG pumped optical parametric oscillator (OPO; Brilliant B/Rainbow, Quantel, France).

Indicative values of excitation energies were obtained by measuring the laser pulse energy behind a cell filled with H_2_O using an energy meter (Gentec QE25SP-H-MB-D0).

For kinetic measurements on the ps/ns and ns/μs timescales (with 2 GHz and 100 MHz bandwidth limits, respectively), the monitoring light was provided by continuous-wave lasers as listed in ref. 13. 2 × 2 × 10 mm cells were used (excitation pulses entered the sample through the 2 × 10 mm window; monitoring light through the 2 × 2 mm window). The monitoring light beams were attenuated by neutral density filters and mechanically chopped to produce a rectangular light pulse of 140 μs duration and energy in the order of 1 μJ at the entrance of the cell, thus avoiding significant actinic effects. This pulse was synchronized with the excitation laser flash (see ref. 27 for more details).

For experiments on millisecond to second timescales, the monitoring light was provided by a tungsten halogen lamp. For selection of a specific wavelength, an interference filter of 5 to 10 nm spectral bandwidth was inserted between the lamp and the sample. A similar filter was placed in front of the detector to block scattered light from the excitation flash and fluorescence. The bandwidth of the amplifier was limited to 30 or 100 kHz.

### Signal analysis

Transient absorption signals were fitted either individually or globally (with shared time constants) using the unweighted Levenberg–Marquardt least-squares minimization algorithm and monoexponential (Fig. 3, 4, 5, 7 (inset), 9, 10, 11 and 13) or biexponential (Fig. 6) decay functions (ExpDec1 and ExpDec2, respectively) in Origin 8.6. A non-zero offset was allowed in the cases where there was a stable residual absorption at the end of the kinetics (due to formation of longer-lived species).

### Quantum yield determination

Quantum yields were determined by comparing the amplitudes of the 457 nm signals from the proteins (at *t* ≈ 10 ns; Fig. 3 and 8) with those yielded by excitation of [Ru(bpy)_3_]Cl_2_ (99.95%)^28^–^30^ under the same geometry and excitation (by 5 ns laser pulses at *λ* = 470 nm). The quantum yield was then calculated using eqn (1):1
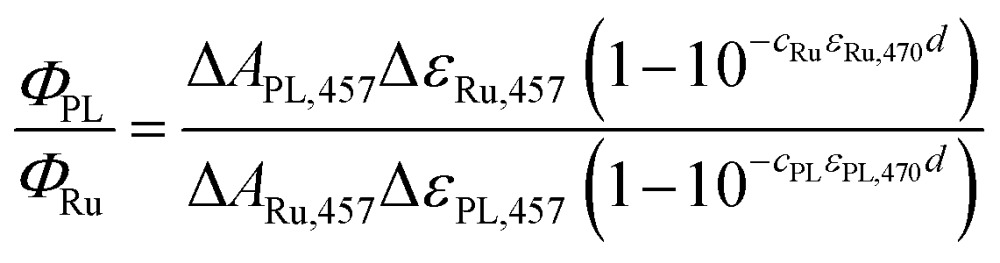
where Δ*A*_457_ is the amplitude of the signals of the photolyase or the Ru complex at *λ* = 457 nm, Δ*ε*_Ru,457_ is the difference of molar absorption coefficients of the metal-to-ligand charge-transfer triplet (^3^MLCT) of the [Ru(bpy)_3_]^2+^ complex and its ground state (∼–11 000 M^–1^ cm^–1^), *c* is the concentration (*c*_Ru_ = 27.4 × 10^–6^ M), *ε*_470_ stands for molar absorption coefficients at the excitation wavelength (*λ* = 470 nm) of the Ru complex (10 128 M^–1^ cm^–1^) or *Mm*CPDII (9460 M^–1^ cm^–1^), and *d* is the optical path of the excitation beam (0.2 cm). Δ*ε*_PL,457_ is the difference in the molar absorption coefficient of the respective photoinduced radical pair and FAD_ox_. The quantum yield of the ^3^MLCT formation (*Φ*_Ru_) equals one (100%).

For oxidized WT *Mm*CPDII, we assume that the observed photoinduced radical pair (at *t* ≈ 10 ns) is predominantly FAD˙^–^ Trp˙, so Δ*ε*_PL,457_ = *ε*_457_(FAD˙^–^) + *ε*_457_(Trp˙) – *ε*_457_(FAD_ox_) = (4740 + 940 – 9205) = –3525 M^–1^ cm^–1^. *c*_PL_ = 40.0 × 10^–6^ M, Δ*A*_PL,457_ = –0.023 and Δ*A*_Ru,457_ = –0.097 then yield *Φ*_PL,WT_ of 55.4%.

For the calculation of the quantum yield of FAD_ox_ photoreduction in the W388F mutant *Mm*CPDII, we assume the vastly prevailing radical pair (at *t* ≈ 10 ns) is FAD˙^–^ Tyr˙ and Δ*ε*_PL,457_ is hence equal to *ε*_457_(FAD˙^–^) + *ε*_457_(Tyr˙) – *ε*_457_(FAD_ox_) = (4740 + 150 – 9205) = –4315 M^–1^ cm^–1^. *c*_PL_ = 38.5 × 10^–6^ M, Δ*A*_PL,457_ = –0.002 and Δ*A*_Ru,457_ = –0.108 then yield *Φ*_PL,W388F_ of 3.7%.

Note that the used value of *ε*_457_(FAD˙^–^) was obtained from the published spectrum^31^ of FAD˙^–^ in an insect cryptochrome. The spectrum of FAD˙^–^ in *Mm*CPDII is not available but it is likely not exactly identical to that in the insect cryptochrome, which could have a certain impact on the accuracy of the calculated quantum yields.

## Results

The primary goal of this study was to characterize the first photoactivation step, *i.e.*, the photoreduction of FAD_ox_ to FAD˙^–^/FADH˙, in the wild-type (WT) class II CPD photolyase (*Mm*CPDII) and to find out how its class-specific tryptophan triad, Trp_381_–Trp_360_–Trp_388_, participates in this process. Our transient absorption spectroscopic data have confirmed the functionality of the tryptophan cascade but they have brought about an additional issue: it turned out that upon electron transfer (ET) to the excited FAD_ox_, one of the oxidized tryptophans (TrpH˙^+^) in the triad undergoes an unprecedentedly fast deprotonation, that is by three orders of magnitude faster than the terminal tryptophans in other PCSf proteins (typically a few hundreds of nanoseconds).^12^,^14^,^19^ This unusually fast rate indicated that the proton is probably transferred to a nearby, structurally-defined acceptor, rather than to disordered bulk solvent.

In order to identify the Trp undergoing the fast deprotonation and the proton acceptor, we have first examined the W388F mutant protein, in which the third (terminal) tryptophan, Trp_388_, was replaced by a redox-inactive phenylalanine, which cannot participate in photoinduced electron transfer to the flavin cofactor. Experiments on this mutant protein unequivocally pointed to the terminal tryptophan Trp_388_ being the fast proton donor and we have hence searched for the likely proton acceptors in its vicinity.

**Fig. 2:**
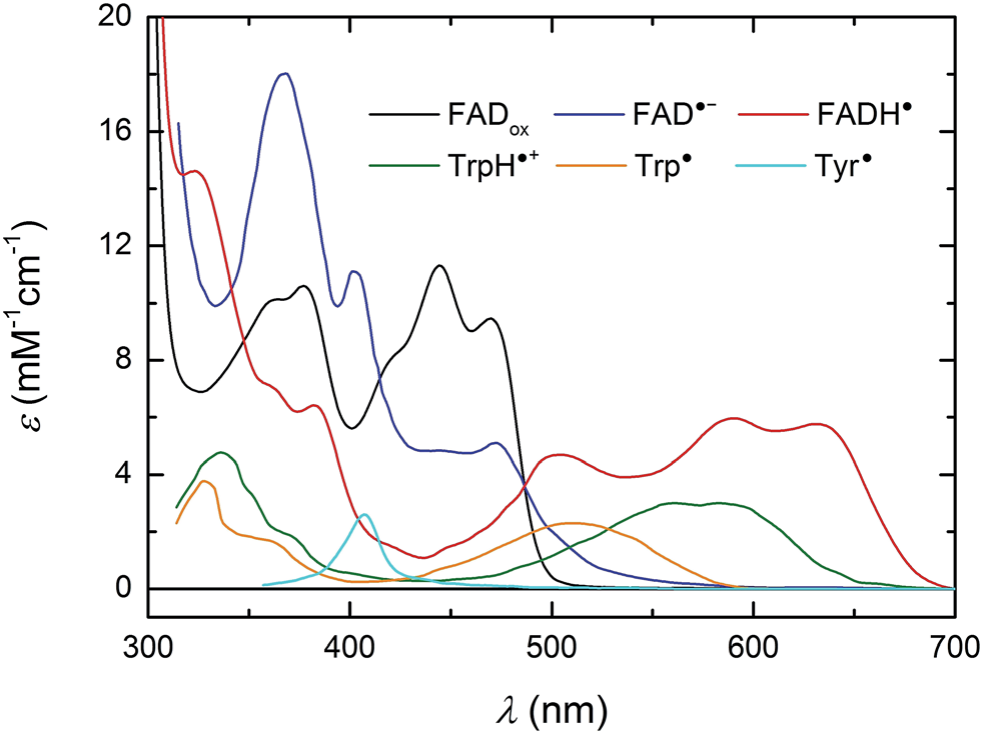
UV/vis spectra of species susceptible to contribute to transient absorption changes following the photoexcitation of FAD_ox_ in *Mm*CPDII. The FAD_ox_ spectrum was measured in *Mm*CPDII and scaled to *ε* (at *λ*_max_) = 11 300 M^–1^ cm^–1^.^44^ The FADH˙ spectrum was constructed as described previously^19^ using the *Mm*CPDII FAD_ox_ spectrum and that of a mixture of FAD_ox_ and FADH˙ in the same sample (obtained by partial photoreduction). The FAD˙^–^ spectrum (from an insect cryptochrome) and spectra of Trp and Tyr radicals are taken from the literature.^31^,^45^,^46^

For a better understanding of the following transient absorption data, absorption spectra of the species that could contribute to changes in transient absorption (FAD_ox_, FAD˙^–^, FADH˙, TrpH˙^+^, Trp˙ and Tyr˙) are shown in Fig. 2.

### Wild-type *Mm*CPDII

Isolated *Mm*CPDII photolyase (WT, as well as all mutants studied here) contains a fully oxidized FAD cofactor (FAD_ox_), which has two pronounced absorption maxima in the near-UV/vis region (see Fig. 2): a double maximum centred around 370 nm and a triple band centred around 445 nm. FAD_ox_ in *Mm*CPDII absorbs up to 500 nm and can hence, in principle, be excited anywhere below this wavelength. For our initial experiments, we have chosen an excitation wavelength of 470 nm (provided by a Nd:Yag-pumped optical parametric oscillator, OPO; pulse duration of ∼5 ns) to avoid interference with our monitoring light sources and to minimize artifacts due to hydrated electrons that are formed upon excitation in the UV.¶
¶Hydrated electrons [with *ε*_max_ (720 nm) ∼18 000 M^–1^ cm^–1^]^47^ are formed upon absorption of a second photon of the excitation pulse by the rapidly formed FAD˙^–^ anion radical. This artefact is much more pronounced in the fast experiments (Fig. 4, 5 and 9) using excitation in the UV because the molar absorption coefficient of FAD˙^–^ at 355 nm is ∼15 000 M^–1^ cm^–1^ (*vs.* ∼5000 M^–1^ cm^–1^ at 470 nm).


**Fig. 3:**
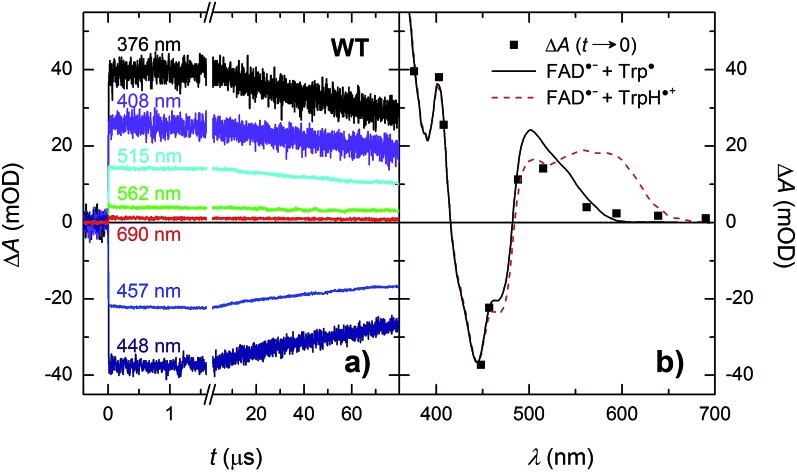
(a) Flash-induced absorption changes on a ns/μs timescale for 40 μM WT *Mm*CPDII at seven characteristic wavelengths (see Fig. S1† for signals at all measured wavelengths). (b) Signal amplitudes at *t* → 0 compared to difference spectra for the formation of FAD˙^–^ + Trp˙ (black solid line) and of FAD˙^–^ + TrpH˙^+^ (red dashed line). The sample was excited at 470 nm by a 5 ns pulse of an energy *E* ∼ 5 mJ per cm^2^. Individual traces are averages of four single flash signals spaced by ∼1 minute.

Based on our previous experience with other members of the PCSf, we have first looked at timescales of a few ns to tens of μs, where we anticipated to observe formation of an FAD˙^–^ TrpH˙^+^ pair and deprotonation of TrpH˙^+^ to Trp˙. Transient absorption changes at representative wavelengths are shown in Fig. 3a, all signals are shown in Fig. S1.† All traces exhibit a step-like increase or decrease, the amplitudes of which (extrapolated to *t* = 0) are represented by black squares in Fig. 3b. If the anticipated FAD˙^–^ TrpH˙^+^ pair were formed, one would expect an absorbance increase below 415 nm and a bleaching between 415 and 485 nm (due to the transformation of FAD_ox_ into FAD˙^–^) combined with an absorption increase between 450 and 650 nm (due to the formation of TrpH˙^+^) – see red dashed line in Fig. 3b. Surprisingly, the signals at 562 and 594 nm (close to the absorption maxima of TrpH˙^+^) and at longer wavelengths exhibited only very small amplitudes compared to the bleaching around 450 nm, excluding the presence of significant amounts of TrpH˙^+^ beyond the given instrument response time (∼5 ns). On the other hand, the amplitudes of the absorption changes could be reasonably described by a difference spectrum reflecting the formation of FAD˙^–^ and a deprotonated tryptophan radical Trp˙ (see Fig. 3b). Assuming the signals are due to 100% FAD˙^–^ Trp˙ pair, the quantum yield of FAD_ox_ photoreduction in the first nanoseconds reaches ∼55% (see the Experimental section for quantum yield determination).

**Fig. 4:**
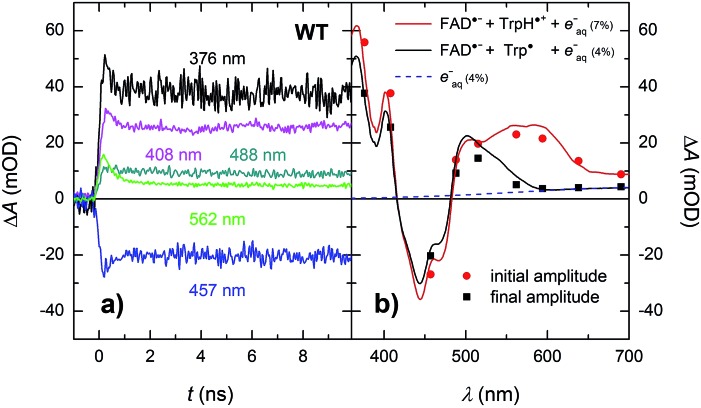
(a) Flash-induced absorption changes on a ps/ns timescale for 64 μM WT *Mm*CPDII at five selected wavelengths (see Fig. S2† for all measured wavelengths). (b) Initial (extrapolated to *t* = 0) and final amplitudes for all measured signals compared to difference spectra for the formation of FAD˙^–^ + TrpH˙^+^ and FAD˙^–^ + Trp˙, respectively, and containing small amounts (7 and 4%, respectively) of hydrated electrons *e*^–^_aq_ (see note ¶ for more details). The sample was excited at 355 nm by a 100 ps pulse of *E* ∼ 5 mJ per cm^2^. The traces are averages of 16 to 64 signals recorded with a repetition rate of 1 Hz.

**Fig. 5:**
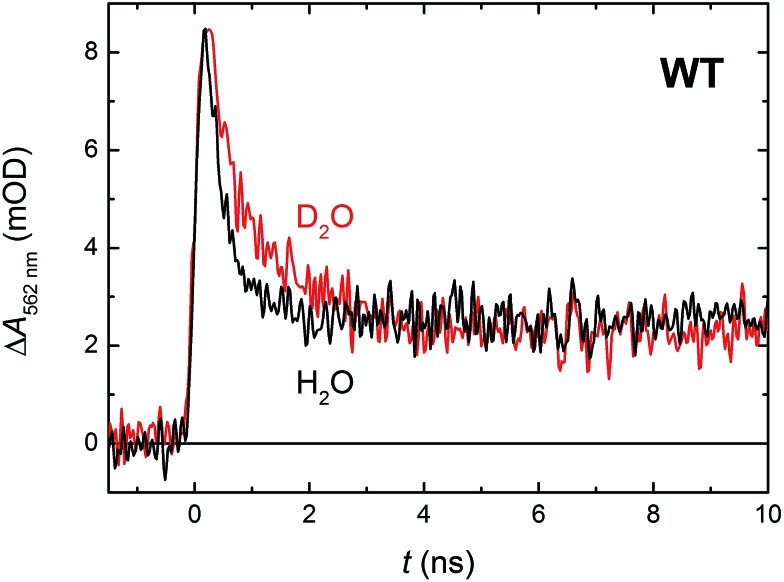
Flash-induced absorption changes at 562 nm showing TrpH˙^+^ deprotonation in 64 μM WT *Mm*CPDII in H_2_O and 51 μM WT *Mm*CPDII in D_2_O (normalized to the amplitude of the signal in H_2_O). Samples were excited at 355 nm by a 100 ps pulse of *E* ∼ 2.5 mJ per cm^2^. The traces are averages of 512 signals recorded with a repetition rate of 1 Hz.

In order to see if we can detect the FAD˙^–^ TrpH˙^+^ pair (precursor of the FAD˙^–^ Trp˙ pair), we have switched to another experimental setup enabling us to access faster timescales.^27^,^28^ To avoid time limitations due to the 5 ns long excitation pulse duration provided by the OPO, we have used a frequency-tripled Nd:YAG laser at 355 nm with 100 ps pulses. The response time of this setup (∼200 ps) has allowed us to observe signals (Fig. 4a and S2†), the initial amplitudes of which were indeed compatible with the presence of an FAD˙^–^ TrpH˙^+^ radical pair (Fig. 4b). The initial amplitudes partially decayed with a time constant of ∼350 ps to yield remaining amplitudes with a difference spectrum consistent with an FAD˙^–^ Trp˙ radical pair (Fig. 4b). We hence attribute the 350 ps phase to deprotonation of TrpH˙^+^ to Trp˙ (∼85%) in competition with FAD˙^–^ TrpH˙^+^ recombination (∼15%; the corresponding partial recovery of FAD_ox_ is well visible, *e.g.*, at 457 nm). Consistent with our attribution to a deprotonation reaction, the kinetics of this phase slows down from ∼350 ps in an H_2_O buffer to ∼800 ps in a D_2_O buffer (Fig. 5), corresponding to a kinetic isotope effect (KIE = *k*_H_/*k*_D_) of 2.3.

**Fig. 6:**
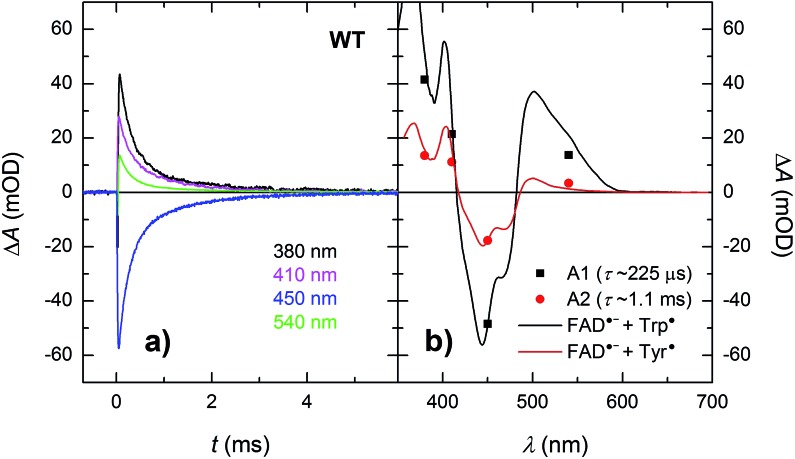
(a) Flash-induced absorption changes on a μs/ms timescale for 44 μM WT *Mm*CPDII. (b) Amplitudes of the two kinetic phases (from a global fit at all four wavelengths) reflecting recombination of the photoinduced radical pairs compared to difference spectra for the formation of FAD˙^–^ + Trp˙ and FAD˙^–^ + Tyr˙, respectively, scaled to the respective amplitudes of the 450 nm signal. The sample was excited at 470 nm by a 5 ns pulse of *E* ∼ 10 mJ per cm^2^. The signals are results of single-flash experiments.

Within the 80 μs experimental time window shown in Fig. 3a, one can observe the beginning of a virtually uniform decay of an FAD˙^–^ Trp˙ radical pair. Using a third setup adapted for monitoring of processes on milliseconds to seconds timescales, we have obtained signals containing the complete decay kinetics (Fig. 6a). It turned out that a biexponential decay function had to be used in order to obtain a good fit, which was an indication of recombination of two distinct pairs of radicals. Global fit of all signals yielded amplitudes for the two processes: those attributed to the faster process (with a time constant of 225 μs and amounting to ∼70% of the total signal amplitude at 450 nm) were indeed consistent with the recombination of FAD˙^–^ Trp˙ pairs but those attributed to the slower process (with a time constant of 1.1 ms and corresponding to the remaining ∼30% of Δ*A*_450 nm_) exhibited a near-zero absorption change at 540 nm, which was incompatible with a tryptophan radical being the recombination partner of FAD˙^–^. When looking at the structure of *Mm*CPDII,^4^ one can notice a tyrosine residue (Tyr_345_) in the vicinity of the terminal tryptophan of the ET chain (3.8 Å edge-to-edge distance from Trp_388_; Fig. 1). An involvement of this tyrosine residue in ET to the excited FAD_ox_ was anticipated, since the mutation of Tyr_345_ to a red-ox inactive phenylalanine was previously shown to slow down the rate of FAD_ox_ photoreduction in a steady-state experiment (compared to the WT protein).^4^ Indeed, the 1.1 ms phase nicely fits the difference spectrum for disappearance of an FAD˙^–^ Tyr˙ pair (Fig. 6b).

Finally, in the absence of external reducing agents, all transient species are completely lost due to recombination within less than 5 milliseconds in the WT *Mm*CPDII (Fig. 6a) and the initial state of the protein with fully oxidized FAD is restored.

**Fig. 7:**
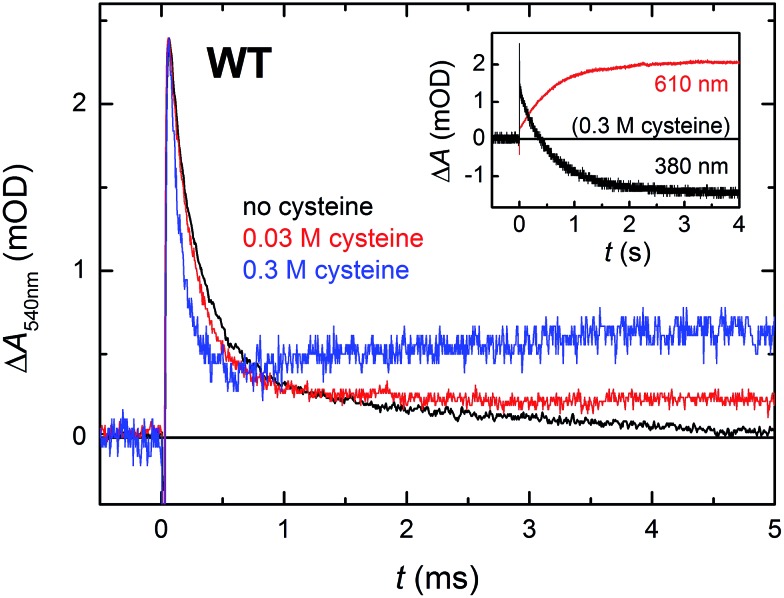
Flash-induced absorption changes at 540 nm (mainly due to Trp˙) on a μs/ms timescale for WT *Mm*CPDII (44.0 μM before dilution by buffer containing cysteine) in the absence and in the presence of cysteine as reducing agent (signals of samples with cysteine are normalized to the amplitude of the cysteine-free sample at *t* → 0 for better visualization of the effect of cysteine on the kinetics of Trp˙ reduction). Reduction of Trp˙ (and Tyr˙) radical(s) by cysteine stabilizes the FAD˙^–^ anion radical, most of which is then protonated to FADH˙ with a time constant of 630 ms. Inset: the disappearance of FAD˙^–^ was observed at 380 nm and the corresponding formation of FADH˙ at 610 nm. Samples were excited at 470 nm by 5 ns pulses of *E* ∼ 2 mJ per cm^2^. Except for the 610 nm trace in the inset, which is an average of 3 signals, all other traces are results of single-flash experiments.

In order for the photoactivation reaction to be efficient, the FAD˙^–^ anion radical has to be stabilized by scavenging of its recombination partner (be it Trp˙ or Tyr˙) by extrinsic reductants. By adding sufficient amounts of cysteine to reduce transiently formed Trp˙ and/or Tyr˙ radicals,^26^ we could compete against the FAD˙^–^ Trp˙/Tyr˙ recombination (Fig. 7; the acceleration of the decay of Δ*A*_540nm_ reflects the reduction of Trp˙ by cysteine; the subsequent rise at 0.3 M cysteine is attributed to protonation of FAD˙^–^, see below) and obtain an isolated metastable FAD˙^–^ radical, which got further stabilized by protonation to yield a neutral FADH˙ radical (see inset of Fig. 7; formation of FADH˙ from FAD˙^–^ is accompanied by a pronounced absorption increase at 610 nm and decrease at 380 nm, see spectra in Fig. 2). Under our experimental conditions (0.01 M Tris–HCl buffer at pH 8.0, 0.1 M NaCl, 10% (v/v) glycerol, 7 °C), this protonation occurred with a time constant of ∼630 ms. However, a closer look at the 540 nm signal with the highest cysteine concentration (0.3 M cysteine; Fig. 7) suggests that, in a small fraction of proteins, FAD˙^–^ can be protonated at a much faster rate, which is reflected by the growth phase (with *τ* ∼ 2.2 ms) following the initial decay of the signal. Judging from the initial amplitude of the signal at 610 nm in the inset of Fig. 7, the fast protonation seems to be possible in ∼15% of proteins (note that only FADH˙ should absorb at 610 nm, contributions from FAD˙^–^ or remaining Trp˙/Tyr˙ radicals are expected to be zero at this wavelength – see Fig. 2).

### W388F mutant lacking the 3^rd^ tryptophan of the triad

**Fig. 8:**
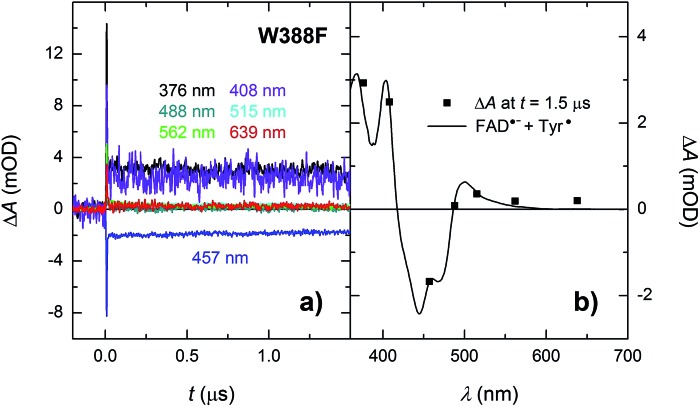
(a) Flash-induced absorption changes on a ns/μs timescale for 38.5 μM W388F mutant *Mm*CPDII at seven characteristic wavelengths. (b) Signal amplitudes at *t* = 1.5 μs are compared to the difference spectrum for the formation of FAD˙^–^ + Tyr˙. The sample was excited at 470 nm by a 5 ns pulse of *E* ∼ 5 mJ per cm^2^. The traces are averages of 8, 16 or 32 signals recorded with a repetition rate of 1 Hz.

In order to find out which of the three tryptophan residues undergoes the unusually fast deprotonation in the WT *Mm*CPDII (Fig. 4 and 5), we decided to examine the behaviour of its W388F mutant, in which the terminal Trp of the triad was replaced by a non-reducing phenylalanine. Our structural data for this and the Y345F mutant (PDB codes ; 5O86, ; 5O8D)^32^ show that there are no compensatory structural changes of residues lining the ET pathway, which could complicate the following analysis and interpretation. The transient absorption signals obtained for the W388F mutant on the ns/μs timescale upon excitation by 5 ns pulses at 470 nm (Fig. 8a) exhibit steep decays in the first nanoseconds, followed by plateaus, the spectral footprint of which corresponds neither to an FAD˙^–^ TrpH˙^+^, nor to an FAD˙^–^ Trp˙ pair, but is compatible with an FAD˙^–^ Tyr˙ pair (Fig. 8b), formed with a quantum yield of mere ∼4% (see the Experimental section for quantum yield determination). The nearest tyrosine to the 2^nd^ Trp of the triad (Trp_360_), which could serve as electron donor to Trp_360_H˙^+^, is Tyr_380_ (4.6 Å edge-to-edge distance; Fig. 1). Tyr_380_ is situated in the vicinity (4.0 Å) of yet another tyrosine Tyr_445_, which is more exposed to solvent and could hence play the role of the terminal electron donor to FAD in the W388F mutant. Alternatively, Trp_360_H˙^+^ could also be reduced by Tyr_345_ (7.1 Å edge-to-edge distance), which is involved in ET in the WT protein.

**Fig. 9:**
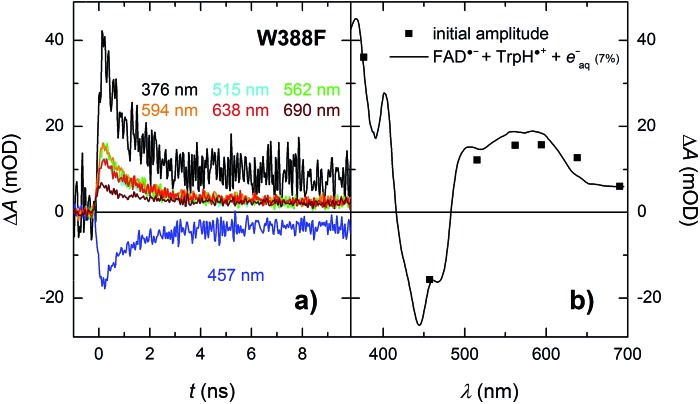
(a) Flash-induced absorption changes on a ps/ns timescale for 55.5 μM W388F mutant *Mm*CPDII at seven characteristic wavelengths. (b) Initial amplitudes (at *t* ∼ 200 ps) of the signals are compared to the difference spectrum for the formation of FAD˙^–^ + TrpH˙^+^ and a small amount (7%) of hydrated electrons *e*^–^_aq_ (see note ¶ for more details). Signals at all wavelengths decay uniformly with a time constant of 1.2 ns. The sample was excited at 355 nm by a 100 ps pulse of *E* ∼ 6 mJ per cm^2^. The signals are averages of 16 to 64 signals recorded with a repetition rate of 2 Hz.

Analogously to the situation in the WT protein, we had to use a different experimental setup to resolve the fast process preceding the formation of the FAD˙^–^ Tyr˙ pair. On the ps/ns timescale, we were able to resolve signals, which were decaying nearly completely with a time constant of ∼1.2 ns (Fig. 9a). The initial amplitudes spectrally fit an FAD˙^–^ TrpH˙^+^ pair (Fig. 9b). We tentatively assign the 1.2 ns decay in the W388F mutant protein to charge recombination in the pair FAD˙^–^ Trp_360_H˙^+^. This recombination presumably competes with a much slower (in the order of 10 to 20 ns) side ET from Tyr_380_ (or Tyr_445_) to Trp_360_H˙^+^, yielding an FAD˙^–^ Tyr˙ pair with a quantum yield of ∼4%.

**Fig. 10:**
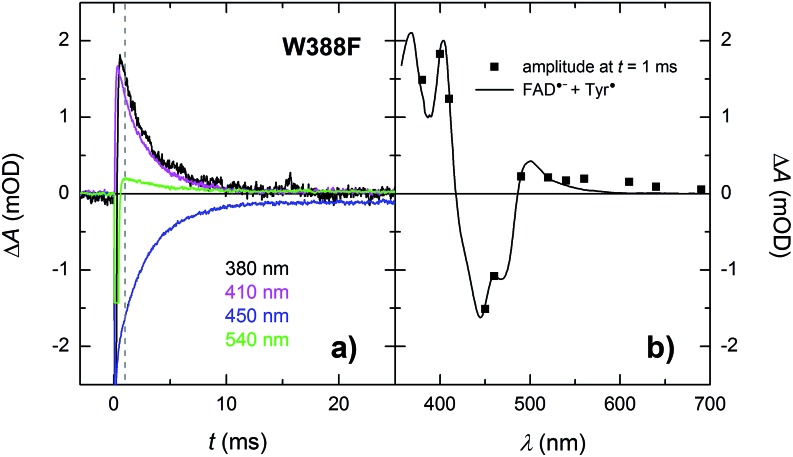
(a) Flash-induced absorption changes on a ms time scale for 21.3 μM W388F mutant *Mm*CPDII at selected wavelengths (for all measured signals, see Fig. S4†). The beginning of some of the signals is distorted by a fluorescence artifact. (b) Signal amplitudes at *t* = 1 ms (*i.e.*, after the end of the fluorescence artifact) are compared with the difference spectrum for the formation of FAD˙^–^ + Tyr˙. All signals exhibit a monoexponential decay with a time constant of 2.7 ms. Sample was excited at 470 nm by a 5 ns pulse of *E* ∼ 10 mJ per cm^2^. The signals are averages of three single-flash experiments spaced by ∼1 minute.

In the absence of extrinsic reducing agents, the FAD˙^–^ Tyr˙ pairs in W388F recombine with a time constant of ∼2.7 ms (Fig. 10), which is ∼2.5× slower than the FAD˙^–^ Tyr˙ recombination in the WT (the observed Tyr˙ radical is hence most probably not Tyr_345_˙ in the W388F mutant *Mm*CPDII). A closer look at Fig. 10b (and also at Fig. 3 and 8) reveals that the amplitudes of signals above 550 nm are not zero, contrary to expectation for an FAD˙^–^ Tyr˙ pair according to the reference spectra shown in Fig. 2. The absorption changes on these timescales can no longer be attributed to hydrated electrons because *e*^–^_aq_ are much shorter-lived. Albeit small, the signals seem to be real and they decay with the same kinetics as the signals at other wavelengths. We hence tentatively attribute the absorption in the red to a particular shape of the FAD˙^–^ spectrum in *Mm*CPDII. Note that FAD˙^–^ spectra with weak absorption in the red have been reported before for some other proteins of the PCSf.^33^,^34^

Like the Trp˙ and Tyr˙ radicals in the WT, the Tyr˙ radical in the W388F mutant could be scavenged in the presence of cysteine and the resulting metastable FAD˙^–^ was further stabilized by protonation to form FADH˙ (see Fig. S3†) at the same rate as in WT *Mm*CPDII under the same conditions (*i.e.*, in ∼630 ms).

### E387Q mutant: searching for an intra-protein proton acceptor

**Fig. 11:**
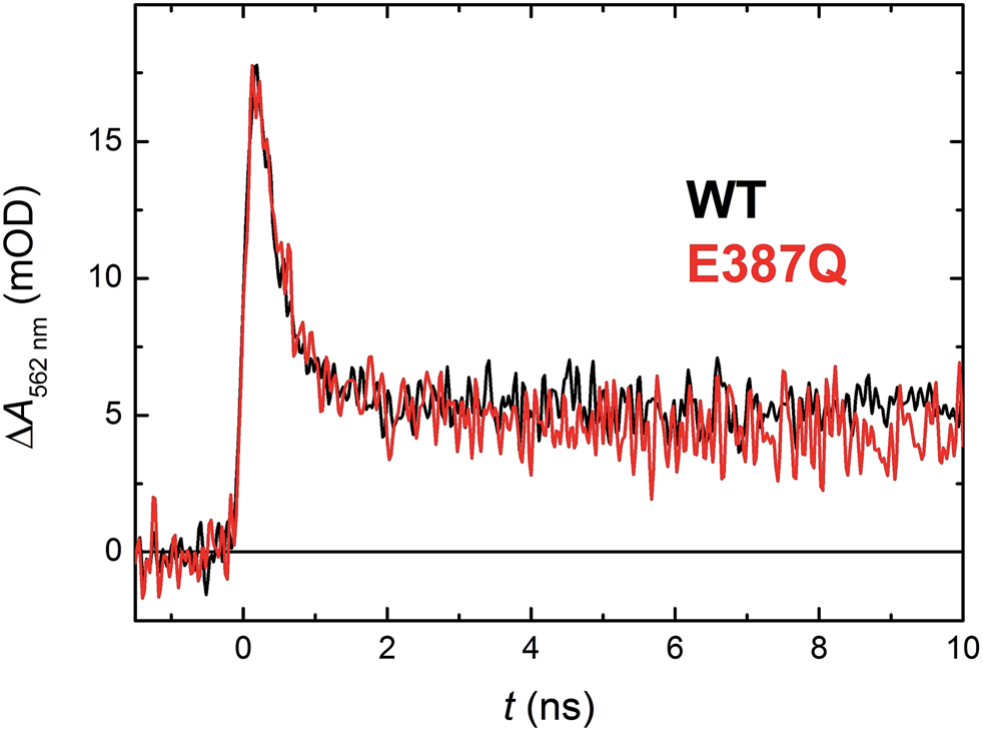
Flash-induced absorption changes at 562 nm showing TrpH˙^+^ deprotonation in 130 μM E387Q mutant *Mm*CPDII in comparison with the WT protein from Fig. 5 (normalized to the amplitude of E387Q). Fitting by a monoexponential decay function gives lifetimes of ∼350 ps for WT and ∼500 ps for E387Q. Both samples were excited at 355 nm by a 100 ps pulse of *E* ∼ 2.5 mJ per cm^2^; the traces are averages of 64 (E387Q) or 512 (WT) signals recorded with a repetition rate of 1 Hz.

**Fig. 12:**
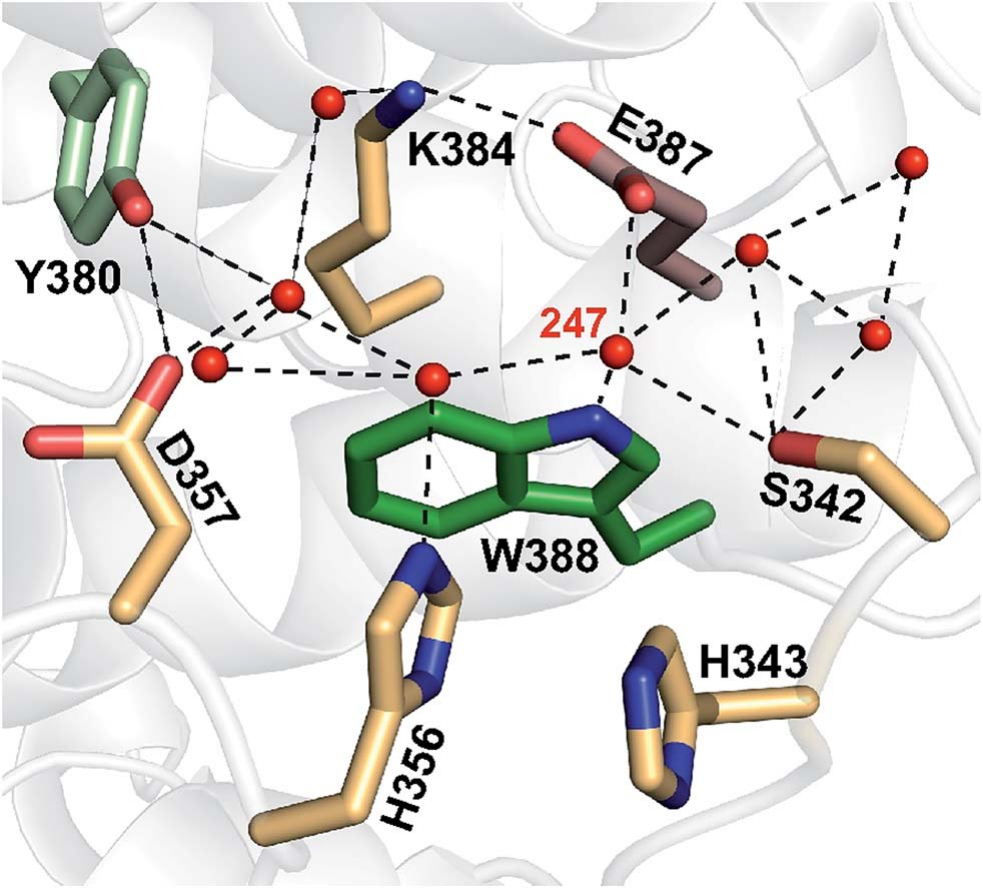
A network of eight water molecules is coordinated by tryptophan 388 and surrounding residues in *Mm*CPDII (PDB entry ; 2XRZ). The water molecule 247 is directly coordinated by the amide group of the tryptophan residue.

According to structural analysis of the environment of the distal tryptophan Trp_388_ in *Mm*CPDII (PDB entry ; 2XRZ), a straight-forward candidate for the intramolecular acceptor of the N1 proton from Trp_388_H˙^+^ is deprotonated‖
‖In order to be a proton acceptor for Trp_388_H˙^+^, Glu_387_ would have to be deprotonated under the given experimental conditions (which is likely at pH 8) and, at the same time, have a higher p*K*_a_ than Trp_388_H˙^+^; note that a typical p*K*_a_ value for a solvent-exposed TrpH˙^+^ is ∼4.^45^,^48^
 Glu_387_ (see Fig. 12): its carboxylic group is as close as 3.6 Å from the N1 atom of Trp_388_. We therefore prepared a mutant, in which the glutamate was replaced by glutamine, which could not serve as a proton acceptor. Fig. 11 compares signals recorded for the WT protein and the E387Q mutant at 562 nm, which is a suitable wavelength for direct monitoring of the deprotonation of TrpH˙^+^ to Trp˙ (and/or its reduction by a tyrosine). The figure clearly shows that the kinetics of TrpH˙^+^ disappearance was almost as fast as in the WT (∼500 ps according to a monoexponential fit in E387Q *vs.* ∼350 ps in WT; a biexponential fit for E387Q yielded ∼400 ps and ∼3 ns at an amplitude ratio of 10 : 1 but the root-mean-square deviation was only marginally better, see Fig. S6†), indicating that Glu_387_ is not the primary proton acceptor. As outlined in the Discussion section, we identified a protein-bound water cluster ideally positioned to serve as the primary proton acceptor.

### Y345F mutant: identification of the tyrosine involved in ET

As mentioned above, there were indications that the tyrosine residue Tyr_345_ also participates in electron transfer to the photoexcited flavin in *Mm*CPDII. We have hence investigated the involvement of this residue in ET by direct comparison of time-resolved spectroscopic signals of the WT and the Y345F mutant *Mm*CPDII.

**Fig. 13:**
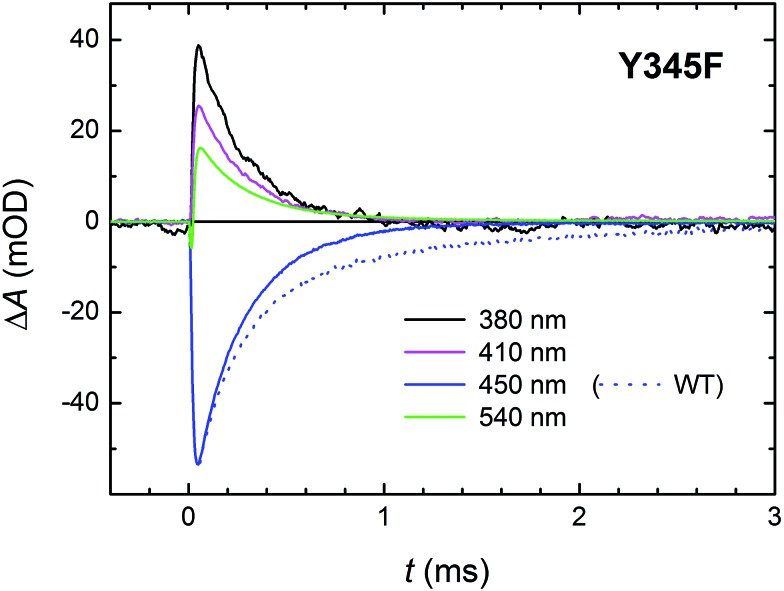
Flash-induced absorption changes on a ms time scale for 70 μM Y345F mutant *Mm*CPDII at selected wavelengths. Unlike in the WT protein (dotted line at 450 nm shown for comparison), signals exhibit a monoexponential decay with a time constant of ∼250 μs. Sample was excited at 470 nm by a 5 ns pulse of *E* ∼ 6 mJ per cm^2^. The signals are averages of four single-flash experiments spaced by ∼1 minute.

Signals in Fig. 13 show a decay of flash-induced absorption changes in the Y345F mutant on the millisecond timescale. Unlike in the WT protein, the signals decay monoexponentially and the slower (1.1 ms) component present in WT (attributed to the FAD˙^–^ Tyr˙ pair and representing approx. 30% of the radical pairs present at the beginning of the ms time scale) is obviously missing. The decay rate of the remaining faster phase (*τ* ∼250 μs) is very close to the value obtained from the fit for the faster process in the WT protein. We conclude that indeed a fraction of tyrosine Tyr_345_ (∼30%) gets oxidized during photoactivation of WT *Mm*CPDII and forms the longest-lived (1.1 ms) radical pair with FAD˙^–^.

With respect to the kinetics and pathway of formation of the tyrosyl radical Tyr_345_˙, we considered two (mutually non-exclusive) possibilities: (i) fast ET from Tyr_345_ to Trp_388_H˙^+^ (in competition with deprotonation of Trp_388_H˙^+^ in 350 ps) and (ii) slow reduction by Tyr_345_ of the deprotonated Trp_388_˙ radical (by proton-coupled ET in competition with recombination of the FAD˙^–^ Trp_388_˙ pair in 225 μs). The similarity of the initial signal amplitude ratios at different wavelengths in the Y345F mutant and the WT protein on the ms time scale (compare Fig. 6a and 13) seems to contradict fast formation of a substantial amount of tyrosyl radical in WT. Nevertheless, we have also compared the signals at 408 nm (wavelength of Tyr˙ absorption maximum) obtained using our fastest experimental setup (Fig. S5†). If the ∼30% of Tyr˙ radical were formed directly from TrpH˙^+^ (in competition with its deprotonation and/or its recombination with FAD˙^–^), the amplitudes of the ∼350 ps decay in the 408 nm signal would have to be clearly different in the WT and in the mutant protein: we would expect that the decay due to ∼15% recombination would be visibly (almost fully) compensated by a growth due to the formation of the Tyr˙ radical in the WT protein. In the Y345F mutant, on the other hand, no Tyr˙ radical can be formed, and the phase should hence reflect pure and uncompensated FAD˙^–^ TrpH˙^+^ recombination (absorption changes due to TrpH˙^+^ deprotonation are negligible at 408 nm; see Fig. 2). Given that there is no significant difference in the ps/ns signals for the two proteins, we can conclude that most of the Tyr˙ radicals are formed later – on the μs timescale in competition with the recombination of FAD˙^–^ with neutral Trp˙.

## Discussion

**Scheme 1:**
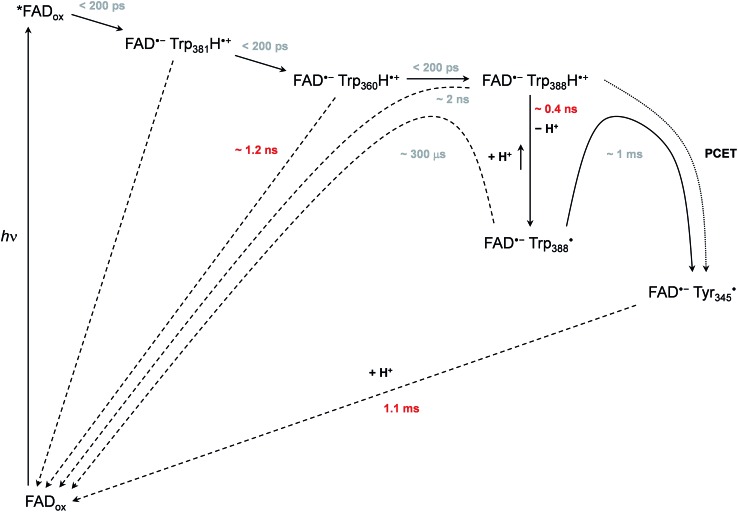
Mechanism of FAD_ox_ photoreduction in the wild-type *Mm*CPDII photolyase inferred from our data. Time constants observed directly are shown in red, time constants in grey are obtained indirectly from branching ratios or estimated (see text and ESI† for more details).

Based on all the data obtained for WT and mutant proteins (in the absence of extrinsic reductants), we have constructed a reaction model (Scheme 1) of the primary processes following FAD_ox_ photoexcitation in WT *Mm*CPDII.

We observed formation of an FAD˙^–^ TrpH˙^+^ pair within our experimental time resolution of 200 ps, followed by deprotonation of TrpH˙^+^ in ∼350 ps, concomitant with ∼15% charge recombination in the FAD˙^–^ TrpH˙^+^ pair (Fig. 4). Assuming direct competition between deprotonation and charge recombination, the intrinsic time constants would be ∼0.4 ns and ∼2 ns, respectively. We assign the deprotonation to Trp_388_, the third (most distant to FAD) member of the triad Trp_381_–Trp_360_–Trp_388_ (Fig. 1), because the deprotonation was lost (and replaced by charge recombination in ∼1.2 ns) in the W388F mutant (Fig. 9).

In analogy to the extensively studied class I CPD photolyase from *E. coli* (*Ec*CPDI), we suppose that ET from Trp_388_ to *FAD_ox_ occurs by ultrafast hopping along the Trp triad also in *Mm*CPDII. Losses may occur due to competition of forward ET with charge recombination in the radical pairs. We have obtained a quantum yield of ∼55% for the formation of the FAD˙^–^ Trp_388_˙ pair (see Results and Experimental section). Taking into account charge recombination in competition with deprotonation of Trp_388_H˙^+^ (see above), the quantum yield of formation of the FAD˙^–^ Trp_388_H˙^+^ pair should be ∼65%, *i.e.*, the same as for the terminal FAD˙^–^ TrpH˙^+^ pair in *Ec*CPDI,^13^ but substantially higher than in a (6-4) photolyase (∼30%)^12^ or in a plant cryptochrome (∼20%).^19^ The particular Trp triad in *Mm*CPDII is hence as efficient in electron transfer as the best known “standard” Trp triad. For the time constants of the hopping steps along the Trp triad, our data can only set an upper limit of ∼100 ps for each of these steps. Given that the edge-to-edge distance between the flavin and the proximal Trp in *Mm*CPDII (4.8 Å) is larger than in *Ec*CPDI (3.7 Å), the first electron transfer step (from Trp_388_H to the excited flavin) is likely to be slower than the 0.8 ps in *Ec*CPDI.^17^ The real kinetics of the electron hopping steps in *Mm*CPDII remain to be resolved by ultrafast methods.

Our data (Fig. 13 and S5†) imply that the tyrosine residue Tyr_345_ (situated 3.8 Å from the third tryptophan of the cascade, Trp_388_) acts as a fourth auxiliary member of the electron-transferring chain, though only about 30% of the Trp_388_H˙^+^/Trp_388_˙ radicals seem to eventually get reduced by Tyr_345_ under our experimental conditions. Because of spectral congestion, we were not able to monitor the kinetics of tyrosine oxidation directly, but from the lack of a significant effect of the Y345F mutation on the ps/ns signals at 408 nm (Fig. S5†) we could conclude that most of Tyr_345_˙ was formed on a slower time scale, *i.e.*, after the deprotonation of Trp_388_H˙^+^. Direct oxidation of Tyr_345_ by Trp_388_˙ seems highly unlikely because it would imply a hydrogen atom transfer over a distance of more than 6 Å. We rather suggest that Tyr_345_ is mostly oxidized by Trp_388_H˙^+^ that is present in a very small amount in thermal protonation equilibrium with Trp_388_˙. Charge recombination in the FAD˙^–^ Trp˙ pair may also proceed *via* thermal reprotonation of the tryptophanyl radical. The observed biphasic recombination kinetics in WT *Mm*CPDII (fitted time constants of 225 μs (∼70%) and 1.1 ms (∼30%); see the Results section and Fig. 6a) can then be described in the framework of Scheme 1 assuming effective time constants of ∼300 μs and ∼1 ms for the charge recombination of FAD˙^–^ Trp_388_˙ and the competing oxidation of Tyr_345_ by Trp_388_˙, respectively, followed by a 1.1 ms recombination of the FAD˙^–^ Tyr_345_˙ pair (see ESI and Scheme S1† for more details).

The μs/ms charge recombination reactions can be blocked (and FAD˙^–^ hence stabilized) by extrinsic electron donors (which are abundant in living cells) that scavenge the Trp˙/Tyr˙ radicals. In this situation, the isolated FAD˙^–^ in *Mm*CPDII becomes protonated in ∼630 ms to form a metastable FADH˙ (Fig. 7). This rate of protonation is slower than the reported ∼200 ms in the *Xenopus laevis* (6-4) photolyase^12^ under similar conditions (0.05 M Tris–HCl buffer at pH 8.3, 0.05 M NaCl, 5% (v/v) glycerol, 10 °C) but faster than the ∼4 seconds observed in the *Escherichia coli* class I CPD photolyase^13^ (0.02 M phosphate buffer at pH 7.5, 0.2 M NaCl, 20% (v/v) glycerol, 7 °C), in spite of a higher pH (8.0) used in the present experiment. The conversion of FADH˙ to the redox state active in DNA repair, *i.e.*, the fully reduced FADH^–^, can follow directly after the absorption of another photon. However, in the presence of oxygen (and in the dark), both FADH˙ and FADH^–^ in isolated *Mm*CPDII spontaneously revert to FAD_ox_ (within a few minutes in an air-saturated solution).

It is a matter of controversy^35^,^36^ whether photoactivation of DNA photolyases through their respective Trp chains is a vital process *in vivo* or whether the FAD cofactor is naturally and always fully reduced in the living cell, but given that at least two such ET chains have evolved independently in the photolyase-cryptochrome superfamily and survived billions of years of evolution, we dare speculate that the efficient photoactivation of photolyases through the Trp (or Trp/Tyr) chains could be important, especially under intense solar irradiation (which causes simultaneously DNA damage and oxidative stress that could potentially deactivate DNA repair by photolyases by oxidation of their FADH^–^). In any case, the electron transfer cascade of three tryptophans and one tyrosine (Trp_381_–Trp_360_–Trp_388_–Tyr_345_ in *Mm*CPDII) is almost strictly conserved within all class II photolyases (Fig. S7†) including plant enzymes like *Os*CPDII from *Oryza sativa*^37^ (Uniprot entry Q0E2Y1; Trp_399_–Trp_378_–Trp_406_–Tyr_363_) or animal orthologs like *Xl*CPDII from *Xenopus laevis* (Q9I910; Trp_470_–Trp_449_–Trp_477_–Tyr_434_).

### Previous observations in the context of our new data

*Mm*CPDII mutant proteins, in which one of the tryptophans of the triad was replaced by non-reducing phenylalanines, were shown to exhibit slower rates of *in vitro* FAD_ox_ photoreduction under steady-state irradiation in the presence of 25 mM dithiothreitol (DTT) as an extrinsic reducing agent.^4^ Mutation of the first Trp of the triad (W381F) had the strongest inhibitive impact and essentially blocked the photoreduction of FAD_ox_. Mutation of the last Trp of the triad (W388F) had the smallest impact, slowing down the FAD_ox_ photoreduction rate by a factor of ∼2 (with respect to the WT protein under the same conditions).^4^ This could seem to be in disaccord with the quantum yield of the FAD˙^–^ Tyr˙ radical pair estimated here (4%), which is substantially lower than the 55% of the FAD˙^–^ Trp˙ pair in the WT, but one has to bear in mind that: (a) the FAD˙^–^ Tyr˙ pair in W388F is longer lived (2.7 ms) than the corresponding FAD˙^–^ Trp˙ and FAD˙^–^ Tyr˙ pairs in the WT (0.3 and 1.1 ms, respectively), which compensates for the lower yield by giving the extrinsic reducing agents more time to scavenge the Tyr˙ radical and stabilize FAD˙^–^ in the W388F mutant, (b) the accessibility of the different radicals to the extrinsic reductants and thereby also the efficiency of the productive encounter of the redox partners is likely to be different, and (c) the systems in the steady-state experiment^4^ were in dynamic equilibria, as the FADH˙ formed by illumination was continuously reoxidized back to FAD_ox_ by molecular oxygen^38^ present in the air-saturated samples. In any case, given the fast forward ET from Trp_388_H to Trp_360_H˙^+^ in WT *Mm*CPDII (<200 ps), the alternative electron transfer pathway involving Tyr_380_ (oxidized by Trp_360_H˙^+^ with an intrinsic time constant of 10–20 ns; see results on W388F), is essentially kinetically switched off and thereby unlikely to play any relevant role in the wild-type protein. By contrast and in spite of the fact, that it reduces only ∼30% of the Trp_388_˙ radicals, the tyrosine Tyr_345_ could noticeably increase the yield of metastable FADH˙, because the lifetime of FAD˙^–^ Tyr_345_˙ (1.1 ms) is almost 5× longer than that of the FAD˙^–^ Trp_388_˙ radical pair (225 μs), which gives more time for exogenous compounds to reduce the recombination partner of FAD˙^–^.

### A network of water molecules mediates fast deprotonation of Trp_388_H˙^+^

With 0.35 ns, deprotonation of the cation radical of the distal member of the Trp triad (Trp_388_H˙^+^) turns out to be three orders of magnitude faster than the corresponding reaction in other studied members of the PCSf: 200 ns in a plant cryptochrome,^19^ 300 ns in *Ec*CPDI,^14^ and 2.5 μs in the *X. laevis* (6-4) photolyase.^12^ The latter rates are comparable to the deprotonation rates in aqueous bulk solution of the transiently formed free TrpH˙^+^ radical either alone (∼700 ns)^39^ or as part of synthetic ruthenium complexes (130–400 ns).^40^,^41^ Accordingly, the very fast deprotonation of the Trp_388_H˙^+^ radical in *Mm*CPDII implies the existence of a structurally defined proton acceptor.

The nearest and most plausible protein-derived candidate for the proton acceptor is Glu_387_, situated 3.6 Å from the deprotonating N1 atom of Trp_388_. Mutation of Glu_387_ had only a minor effect on the deprotonation kinetics, excluding it as the direct proton acceptor. A closer look at the crystal structure shows that N1 of the Trp_388_ indole ring forms an H-bond to the water molecule 247 (*d*_N1–O_ = 3.4 Å). This water is ideally positioned to function as initial proton acceptor from Trp_388_H˙^+^ by being part of a network of eight surface-bound water molecules, which are further coordinated by Tyr_380_, Lys_384_, Glu_387_, Ser_342_, His_356_ and Asp_357_ (Fig. 12). Superposition of the archaeal *Mm*CPDII structure (PDB entry ; 2XRZ) with the only other structurally characterized class II photolyase, the eukaryotic *Os*CPDII (; 3UMV), shows a preserved arrangement of the water network (Fig. S7†). Seven water molecules were found close to the distal *Os*CPDII Trp_406_ that is surrounded by a similar motif (Tyr_398_, Lys_402_, Glu_405_, Glu_360_, His_361_, His_374_ and Asp_375_). This structurally defined water network and its arrangement appear to be unique to class II CPD photolyases, because in the known crystal structures of members from all other PCSf sub-families the distal tryptophans lack H-bonding to other residues or well defined water molecules. When analysing the sequence divergence of these residues among class II photolyases (Fig. S7†), it is appealing that over a half of them (Trp_388_, Tyr_380_, Lys_384_, Glu_387_ and Asp_357_ in *Mm*CPDII numbering) are highly conserved.

Given the generally short residence times of first shell water molecules on protein surfaces (20–200 ps),^42^ the off-diffusion of the proton from the site next to Trp_388_ may be facilitated by exchange reactions with the bulk. Alternatively, the water network may transfer the proton *via* a Grotthuss-like mechanism to bulk water or an unknown final acceptor along the protein surface. In any case, our findings suggest that the unusually fast (water cluster-mediated) deprotonation of the terminal tryptophan is a general property of all class II CPD photolyases.

Protein-bound water molecules and their dynamics have been shown to be essential for proton transport and biological function of other proteins such as bacteriorhodopsins.^43^ Based on our findings, class II photolyases add to biological systems, in which water clusters play an important role in proton transfer. Further insight may be gained by simulations of the dynamics of the water cluster following the formation of the cation radical Trp_388_H˙^+^ in *Mm*CPDII.

## Conflicts of interest

There are no conflicts of interest to declare.

## Supplementary Material

Supplementary information
