# Supplementary material for: Sub-nanosecond tryptophan radical deprotonation mediated by a protein-bound water cluster in class II DNA photolyases
Source: Chem Sci. 2017 Dec 11;9(5):1200–12. doi: 10.1039/c7sc03969g (PMC5885780; doi:10.1039/c7sc03969g)
Supplement: Supplementary file 1 [file SC-009-C7SC03969G-s001.pdf]

Electronic Supplementary Material (ESI) for Chemical Science.
This journal is © The Royal Society of Chemistry 2017

Electronic Supplementary Information

Sub-nanosecond tryptophan radical deprotonation mediated by a protein-bound water cluster in class II DNA photolyases

Pavel Müller,*‡a Elisabeth Ignatz,‡b Stephan Kiontke,¶b Klaus Brettel[a] and Lars-Oliver Essen*[b]

[a] Institute for Integrative Biology of the Cell (I2BC), CEA, CNRS, Univ. Paris-Sud, Université Paris-Saclay, 91198, Gif-sur-Yvette cedex, France. *E-mail: pavel.muller@i2bc.paris-saclay.fr

[b] Department of Chemistry, LOEWE Center for Synthetic Microbiology, Philipps University, 35032 Marburg, Germany. *E-mail: essen@chemie.uni-marburg.de

¶ Present address: Structural Biology, FB5 Biology/Chemistry, University of Osnabrück, 49076 Osnabrück, Germany.

‡ The authors contributed equally to this work.

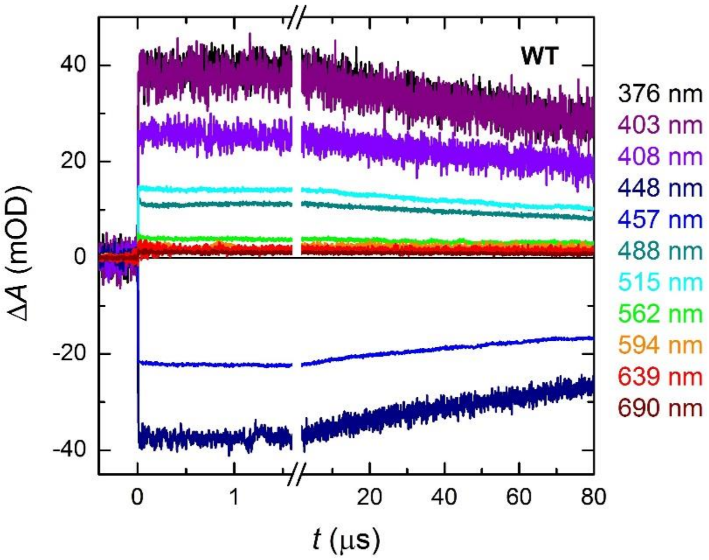

Fig. S1 Flash-induced absorption changes on a ns/μs time scale for WT *Mm*CPDII at all measured wavelengths. Experimental conditions are described in the legend of Fig. 3.

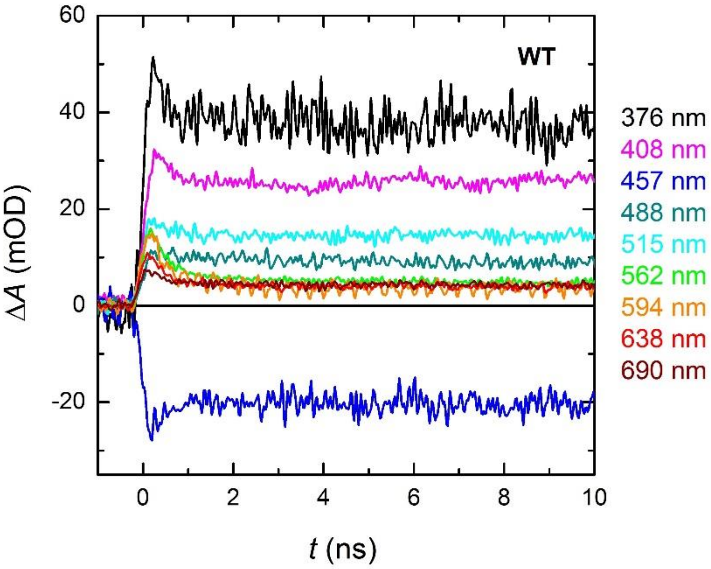

Fig. S2 Flash-induced absorption changes on a ps/ns time scale for WT *Mm*CPDII at all measured wavelengths. Experimental conditions are described in the legend of Fig. 4.

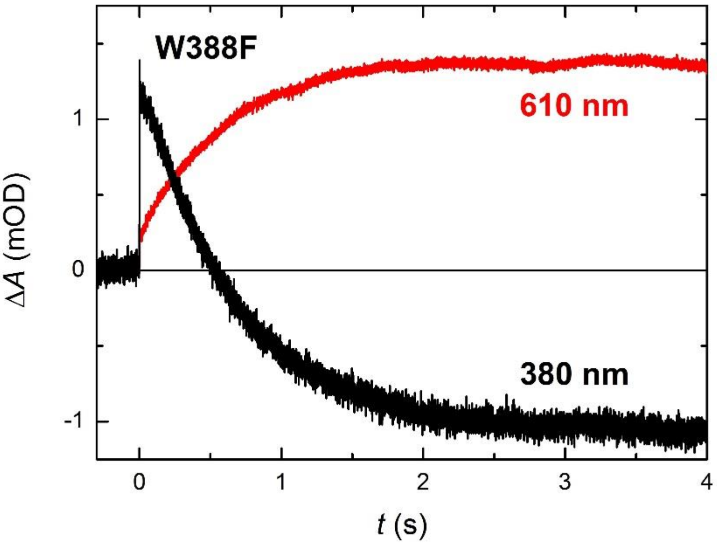

Fig. S3 Flash-induced absorption changes on a seconds time scale for 20.9 μM W388F mutant *Mm*CPDII (in the presence of 10 mM cysteine) reflecting the slow (630 ms) protonation of $FAD^{\bullet-}$ to $FADH^{\bullet}$. The sample was excited at 470 nm by a 5 ns pulse of E ~10.0 mJ per cm^2. The signals are averages of three single-flash experiments spaced by ~1 minute.

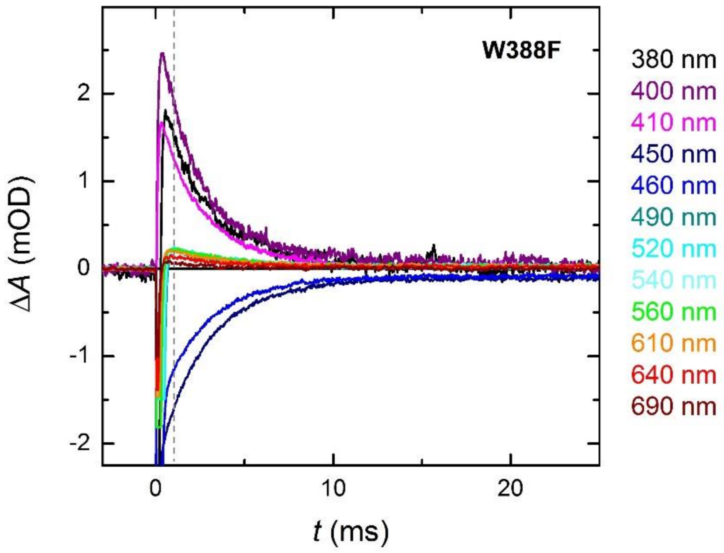

Fig. S4 Flash-induced absorption changes on a ms time scale for W388F mutant *Mm*CPDII at all measured wavelengths. Experimental conditions are described in the legend of Fig. 10.

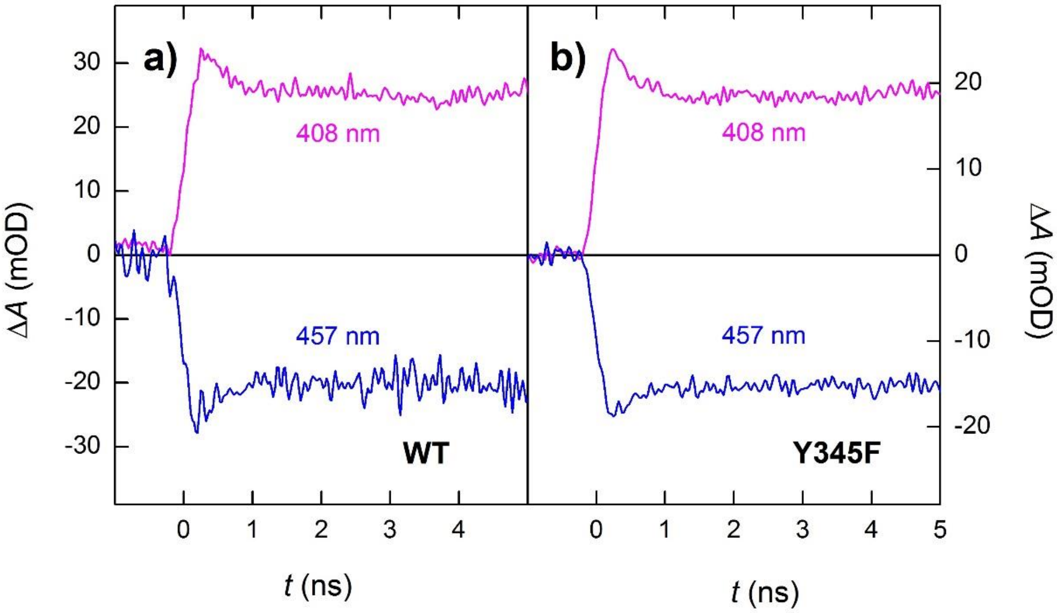

Fig. S5 Comparison of the flash-induced absorption changes on a ps/ns time scale for a) WT *Mm*CPDII (64 μM) and b) its Y345F mutant (69 μM). The samples were excited at 355 nm by 100 ps pulses of *E* ~5 mJ per cm^2 (WT) or ~4 mJ per cm^2 (Y345F).

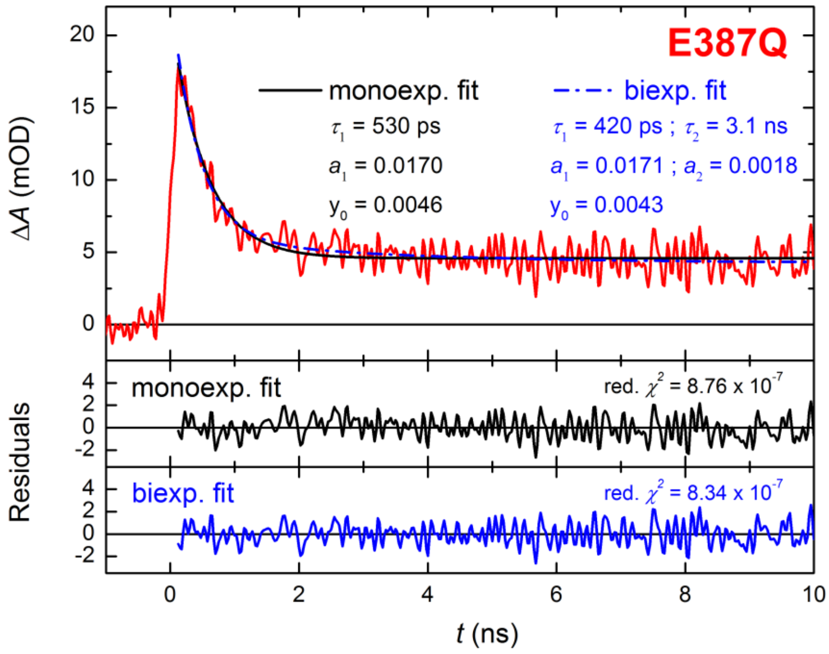

Fig. S6 Comparison of mono- and biexponential fits of the flash-induced absorption changes at 562 nm in E387Q *Mm*CPDII (identical to the signal shown in Fig. 11 of the main text). Fit functions: $\Delta A(t) = a_1 e^{-\frac{t}{\tau_1}} + y_0$ (black solid line) and $\Delta A(t) = a_1 e^{-\frac{t}{\tau_1}} + a_2 e^{-\frac{t}{\tau_2}} + y_0$ (blue dashed line).

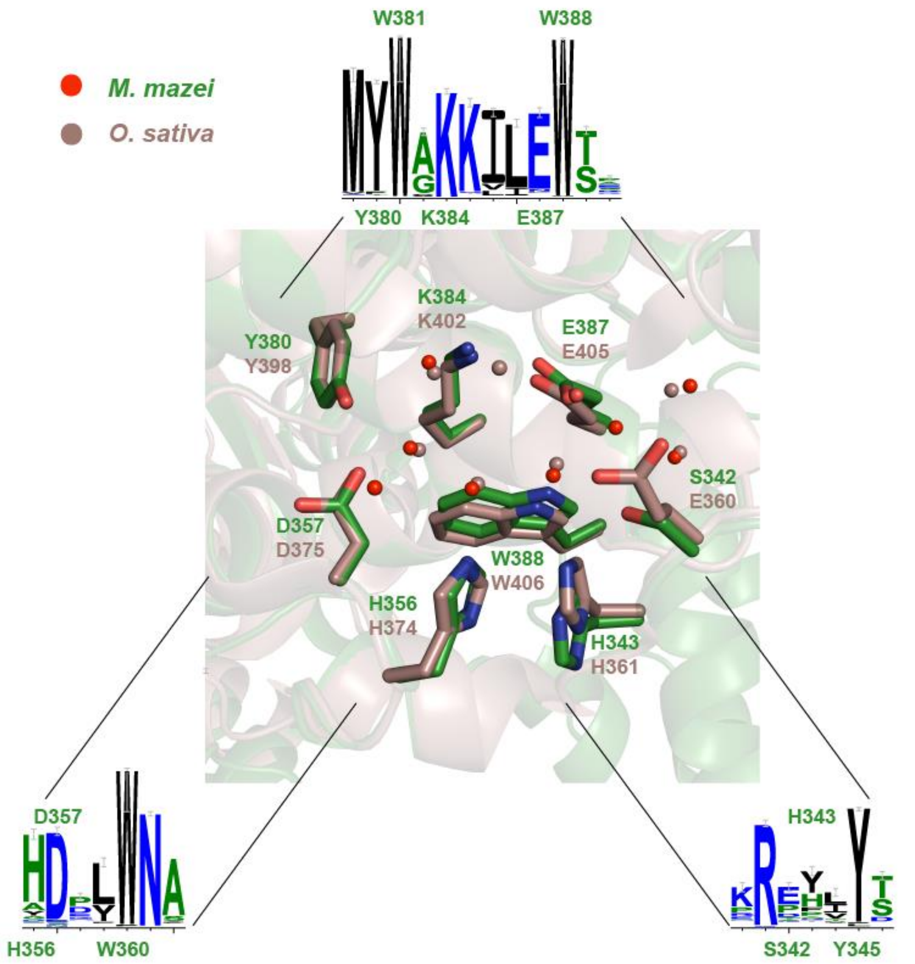

Fig. S7 The immediate environment of the terminal tryptophan and the water network participating in its deprotonation during photoactivation: superposition of two crystal structures of class II CPD photolyases from the archaeon *Methanosarcina mazei* (PDB entry 2XRZ)[1] and the plant *Oryza sativa* (PDB entry 3UMV)[2]. The degree of conservation of the individual amino acids determined from 451 non-redundant class II photolyase sequences is proportional to the height of the corresponding symbols (W360, W381 and W388 are strictly conserved).

Determination of intrinsic rate/time constants of FAD$^{\bullet-}$ Trp$_{388}^{\bullet}$ recombination, competing ET from Tyr$_{345}$ to Trp$_{388}^{\bullet}$ and FAD$^{\bullet-}$ Tyr$_{345}^{\bullet}$ recombination for the Scheme 1 in the main text

To describe the reaction kinetics following the formation of FAD$^{\bullet-}$ Trp$_{388}^{\bullet}$, we used the following simplified scheme neglecting the very low population of FAD$^{\bullet-}$ Trp$_{388}$H$^{\bullet+}$ in protonation equilibrium with Trp$_{388}^{\bullet}$):

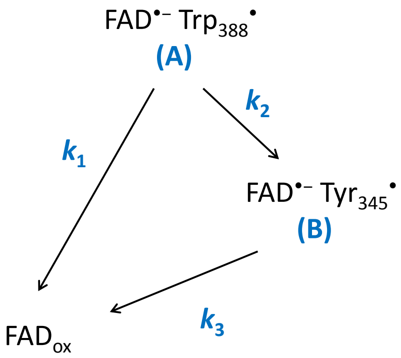

Scheme S1

Scheme S1 is described by two coupled differential equations:

$$\frac{d[\mathrm{A}]}{dt} = -(k_1 + k_2)[\mathrm{A}] \qquad (1) \qquad\qquad \frac{d[\mathrm{B}]}{dt} = k_2[\mathrm{A}] - k_3[\mathrm{B}]. \qquad (2)$$

Initially, only the state A is populated, *i.e.*, [A](t=0) = [A]$_0$ = 1 and [B](t=0) = 0.

Solving these equations for [A] and [B] gives:

$$[\mathrm{A}](t) = [\mathrm{A}]_0 e^{-(k_1+k_2)t} \qquad (3)$$

$$[\mathrm{B}](t) = [\mathrm{A}]_0 \frac{k_2}{k_1+k_2-k_3}\left(e^{-k_3 t} - e^{-(k_1+k_2)t}\right) \qquad (4)$$

Using for the absorption change

$$\Delta A(t) = \left(\Delta\varepsilon_{\mathrm{A}}[\mathrm{A}](t) + \Delta\varepsilon_{\mathrm{B}}[\mathrm{B}](t)\right)d, \qquad (5)$$

where d is the optical path length, one obtains

$$\Delta A(t) = a_1 e^{-(k_1+k_2)t} + a_2 e^{-k_3 t} \qquad (6)$$

with

$$a_1 = \left(\Delta\varepsilon_{\mathrm{A}} - \Delta\varepsilon_{\mathrm{B}} \frac{k_2}{k_1+k_2-k_3}\right)[\mathrm{A}]_0 d \quad \text{and} \quad a_2 = \frac{k_2}{k_1+k_2-k_3}\Delta\varepsilon_{\mathrm{B}}[\mathrm{A}]_0 d.$$

$$\frac{a_1}{a_2} = \frac{k_1+k_2-k_3}{k_2}\frac{\Delta\varepsilon_{\mathrm{A}}}{\Delta\varepsilon_{\mathrm{B}}} - 1 \qquad (7)$$

Eq. 6 consists of two terms corresponding to the two fitted phases. The pre-exponential factors reflect the relative amplitudes (a_1 and a_2) of the respective phases, the rates of the phases are described by the constants in the exponents: the sum (k_1 + k_2) determines the fast phase (disappearance of A and

the concomitant formation of B) and k_3 determines the slow phase (disappearance of B). The sum ($k_1 + k_2$) and $\boldsymbol{k_3}$ and are obtained directly from the fit: $k_1 + k_2$ = 4444 s^{-1} (1/225x10^{-6}s) and $\boldsymbol{k_3}$ = **909** $\mathbf{s^{-1}}$ (1/1.1x10^{-3}s), respectively.

To obtain k_1 and k_2 individually, we additionally used the amplitude ratio a_1/a_2 = 7/3 observed at 450 nm, where the $FAD^{\bullet-}/FAD_{ox}$ contribution largely prevails over those of $Trp^{\bullet}$/TrpH and $Tyr^{\bullet}$/TyrH (Fig. 2).

Using eq. 7 with $\Delta\varepsilon_A/\Delta\varepsilon_B$ = 0.923 (Fig. 2), one obtains:

$$\boldsymbol{k_2} = \frac{(k_1 + k_2 - k_3)\,\frac{\Delta\varepsilon_A}{\Delta\varepsilon_B}}{\frac{a_1}{a_2} + 1} = \frac{(4444 - 909)\;0.923}{\frac{7}{3} + 1}\,\mathrm{s}^{-1} = \mathbf{979\ s^{-1}}$$

$$\boldsymbol{k_1} = (4444 - 979)\mathrm{s}^{-1} = \mathbf{3465\ s^{-1}}$$

The intrinsic rate constants k_1, k_2 and k_3 convert into time constants $\boldsymbol{\tau_1}$, $\boldsymbol{\tau_2}$ and $\boldsymbol{\tau_3}$ of **~300 µs**, **~1ms** and **1.1 ms**, respectively, which are indicated in the **Scheme 1** of the main text.

References

1. S. Kiontke, Y. Geisselbrecht, R. Pokorny, T. Carell, A. Batschauer and L.-O. Essen, *EMBO J.*, 2011, **30**, 4437-4449.
2. K. Hitomi, A. S. Arvai, J. Yamamoto, C. Hitomi, M. Teranishi, T. Hirouchi, K. Yamamoto, S. Iwai, J. A. Tainer, J. Hidema and E. D. Getzoff, *J. Biol. Chem.*, 2012, **287**, 12060-12069.
